# Immunotherapy in Thoracic Malignancies: New Treatment and New Hope

**DOI:** 10.3390/curroncol29020070

**Published:** 2022-02-02

**Authors:** Barbara Melosky

**Affiliations:** BC Cancer, Vancouver Centre, 600 West 10th Avenue, Vancouver, BC V5Z 4E6, Canada; bmelosky@bccancer.bc.ca

Over these last two pandemic years, we have all experienced profound changes in how we practice, how we work, and how we live. Many of our patients have experienced changes in the way that their cancer care and their treatments were delivered as we adjusted to social distancing measures. Our nurses and staff had to work extra hard to accommodate these changes and to ensure our patients received the best care possible under altered circumstances. Unfortunately, cancer’s work was not delayed or cancelled, and people have continued to be diagnosed with lung cancer. In this Special Issue, titled “Current State of Immunotherapy for Lung Cancer”, our goal was to provide a comprehensive summary of the state of the art of immunotherapy for this deadly disease.

We are finally identifying and validating appropriate immunotherapy biomarkers for lung cancer. For many years we struggled with understanding the prognostic implications of PD-L1 levels and tumor mutational burden (TMB), and how this will influence therapy. Khwaja and Chu [1] describe the role of PD-L1 expression, tumor mutational burden (TMB), smoking history, STK11/KEAP1 mutations, and the gut microbiome as predictive markers to immunotherapy benefit, as defined by overall response and median overall survival.

The immunotherapy biomarker we all use is PD-L1 expression levels. As multiple antibodies exist and the definitions of expression levels vary, decision making can be challenging. The pathologist plays an essential role in characterizing PD-L1 expression, which influences patient therapy. Jöhrens and Rüschoff [2] explore the role of the pathologist in PD-L1 testing, and emphasize the importance of selecting deliberate controls, careful validation, necessary preanalytics, and appropriate internal and external quality assessment.

Immunotherapy in the first line setting of advanced non-small cell lung (NSCLC) is complex and is becoming crowded. Options include single-agent immunotherapy, immunotherapy combined with chemotherapy, dual immunotherapy, and dual immunotherapy plus chemotherapy, often depending on the PD-L1 level of expression. Patients with wild-type NSCLC can be divided into three major classes based on PD-L1 expression levels, including those with high (> 50%), moderate (1–49%), and negative PD-L1 expression. Denault and Melosky [3] describe clinical recommendations for our patients, including those who have never smoked.

Immunotherapy is becoming increasingly nuanced, as new combinations are being used to augment our immune responses to tumors. The tumor immune microenvironment (TIME) environment, where tumor and immune cells interact, is crucial for determining therapeutic outcomes. Corke and Sacher [4] explore TIME and more. Targeting co-stimulatory molecules, including CD137, CD134, CD278, are described. Priming strategies with radiation, oncolytic viruses, and STING and TLR agonists may potentially change practice. The role of targeted therapy, VEGF, and the TGF-β signaling pathway are reviewed. Finally, adoptive cell therapy is discussed.

Immunotherapy has shown survival advantages in other stages and thoracic malignancies. For patients with resected early stage NSCL, adjuvant chemotherapy was the only treatment option. Although a survival benefit exists, the absolute benefit of adjuvant chemotherapy is small. Both adjuvant and neoadjuvant immunotherapy promise to change outcomes for these patients. Cao et al. [5] present a meta-analysis of neoadjuvant immunotherapy for patients with resected early stage NSCLC. Eighteen publications from sixteen different studies were analyzed and demonstrate that this approach is feasible and safe.

Perdrizet and Cheema [6] review the use of immunotherapy for patients with Stage III NSCLC. The use of durvalumab as consolidative therapy after chemotherapy and radiation in unresectable stage III has now become a standard of care globally. Unanswered questions of timing (sequential versus concurrent treatment), different combinations of immune inhibitors, and biomarkers that may predict response are explored.

The treatment of extensive-stage small cell (ES-SCLC) was unchanged for decades. El Sayed and Blais [7] review the results of the IMpower-133 and the CASPIAN trials which disrupted the treatment landscape in extensive-stage small cell lung cancer. Finding a biomarker to aid decision making has been challenging. This paper highlights the complexity and the promise of subtyping this patient group.

Similarly, previous treatment options for patients with mesothelioma were disappointing and stagnant. Banerji et al. [8] review the role of immunotherapy in the treatment of malignant mesothelioma. The CheckMate 743 trial demonstrated that the combination of ipilimumab and nivolumab is superior to cisplatin doublet. This is great news for our patients. Trials using novel strategies of immunotherapy in combination with chemotherapy and dendritic cell therapy (DCT) and chimeric antigen receptor (CAR-T) cell therapy are reviewed and are awaited with enthusiasm.

Finally, immune-related adverse events must always be considered. In the feature paper of this Special Issue, Coschi and Juergens [9] present an article appropriately called “The price of success”. The mechanism of action of immunotherapy toxicity is explained. Organ-specific immune-related adverse events including dermatologic, endocrine, gastrointestinal, lung, renal and neurologic sites are described. Treatment of toxicity with steroids often leads to immunosuppression; immunotherapy re-challenge and efficacy in this clinical context is discussed. This review is an essential read for the treating clinician.

It is not possible for us to discuss the treatment innovations and therapeutic advancements that have occurred for our patients without acknowledging the support of the many companies operating in this space. We wish to thank Amgen Canada, AstraZeneca Canada Inc, Eisai Canada Limited, Hoffman La Roche Canada (journal publication fees only), Jazz Pharmaceuticals Canada Inc., Novartis Canada, Sanofi Canada, and Pfizer Canada Inc. for providing funding to pay journal publication fees and honorariums for some of the publications in this Special Issue, as well as administrative support. These entities did not influence the content of the articles, nor did they review the article prior to publication.

This Special Issue summarizes the current state of immunotherapy in lung cancer. Our use of immunotherapy will continue to evolve as we start to use this treatment modality to treat all stages and different histologies of thoracic malignancy. We look forward to seeing how immunotherapy will continue to improve survival for all of our patients.
